# Dietary Pattern Extraction Using Natural Language Processing Techniques

**DOI:** 10.3389/fnut.2022.765794

**Published:** 2022-03-09

**Authors:** Insu Choi, Jihye Kim, Woo Chang Kim

**Affiliations:** ^1^Department of Industrial and Systems Engineering, Korea Advanced Institute of Science and Technology, Daejeon, South Korea; ^2^Department of Genetics and Biotechnology, College of Life Sciences, Kyung Hee University, Yongin, South Korea

**Keywords:** dietary pattern, machine learning, natural language processing (NLP), word embedding, topic modeling

## Abstract

In this study, we observed the changes in dietary patterns among Korean adults in the previous decade. We evaluated dietary intake using 24-h recall data from the fourth (2007–2009) and seventh (2016–2018) Korea National Health and Nutrition Examination Survey. Machine learning-based methodologies were used to extract these dietary patterns. Particularly, we observed three dietary patterns from each survey similar to the traditional and Western dietary patterns in 2007–2009 and 2016–2018, respectively. Our results reveal a considerable increase in the number of Western dietary patterns compared with the previous decade. Thus, our study contributes to the use of novel methods using natural language processing (NLP) techniques for dietary pattern extraction to obtain more useful dietary information, unlike the traditional methodology.

## Introduction

Diets are affected by interactions between biological, social, economic, and cultural factors (1). Therefore, people's diet can eventually change. In particular, dietary patterns have rapidly evolved in South Korea due to this country's early westernization compared with most Asian countries (2). The transition of dietary patterns should be monitored because it holds an important risk factor for developing chronic diseases. Traditional methods have been employed to show unique dietary patterns or observe changes in dietary patterns over time in Korean populations (3, 4).

However, epidemiological studies on dietary pattern extraction using traditional methods have many limitations. For example, many subjective decisions are included in food grouping for extracting dietary patterns. Moreover, obtaining information on particular food items has become difficult due to their broad classification.

Therefore, this study applies natural language processing (NLP) techniques to overcome these constraints. NLP techniques have been widely used in many areas. Recently, they were applied in the nutritional domain. Several papers use NLP techniques to analyze their nutrition goals. Tamaddoni-Nezhad et al. (5) demonstrated that logic-based machine learning methods and NLP techniques can be employed to generate food webs from ecological census data. Zhu et al. (6) attempted to obtain a food collocation and investigate its effect using NLP preprocessing methodologies. Korpusik and Glass (7) proposed a novel approach to food journaling that uses speech and language understanding technology to achieve an efficient self-assessment of energy and nutrient consumption. Tao et al. (8) introduced an overview of data sources, computational methods, and applications of text data in the food industry. In addition, Van Erp et al. (9) discussed a food concept and researched on recipes to solve health and sustainability issues addressed in an interdisciplinary manner. They integrated NLP and other AI techniques with historical food research, such as food science and nutrition. Bakhtin et al. (10) presented a text mining study on science and technology in food production from more than 30 million documents. They proposed a methodology that demonstrates the future of food production with each new data that become available and served as an early warning system for a changing technology landscape.

However, studies on modeling-based analysis for food intake data rarely exist. Therefore, we aimed to extract the dietary patterns of Korean adults using NLP techniques and observe the changes in their dietary patterns over the last decade from large-scale national representative data. The remainder of this paper is organized as follows. In Section “Data and Methodology,” the methods for data collection and NLP techniques used to analyze them are presented. In Section “Results,” the main results of our study are described. The main results of this study are discussed in Section “Discussion.” Finally, the concluding remarks are presented in Section “Conclusion.”

## Data and Methodology

### Data

This study used data collected from the Korea National Health and Nutrition Examination Survey (KNHANES) conducted by the Korea Disease Control and Prevention Agency. KNHANES is a clustered, multistage, and stratified sampling design that annually assesses the diet and health of the Korean population. Many studies on the diet and nutrition of the Korean population use this dataset owing to its representation and credibility (4, 11, 12). We used the 2007–2009 and 2016–2018 data from the fourth and seventh KNHANES, respectively. A total of 16,187 and 16,809 study participants were from the fourth and seventh KNHANES datasets, respectively. The demographic characteristics of the study participants are summarized in Table 1.

**Table 1:** Characteristics of survey participants.

**Category**	**2007–2009**	**2016–2018**
All	16,187	16,809
**Sex**		
Male	6,592 (40.7)	7,144 (42.5)
Female	9,595 (59.3)	9,665 (57.5)
**Age**		
19–39	4,643 (27.5)	5,296 (32.7)
40–59	6,189 (36.8)	5,814 (35.9)
60+	5,997 (35.7)	5,077 (31.4)
**House type**		
General	9,750 (60.2)	7,831 (46.6)
Apartment	6,437 (39.8)	8,978 (53.4)
**Highest level of education**		
Elementary School	4,589 (28.3)	3,288 (19.6)
High School	6,830 (42.2)	6,342 (37.8)
Over Associate Degree/Bachelor Degree	3,666 (20.7)	5,639 (33.5)

*The difference between the total number of respondents and the number of respondents per category means no response*.

### Methodology

#### Data Preprocessing

**Figure 1:**
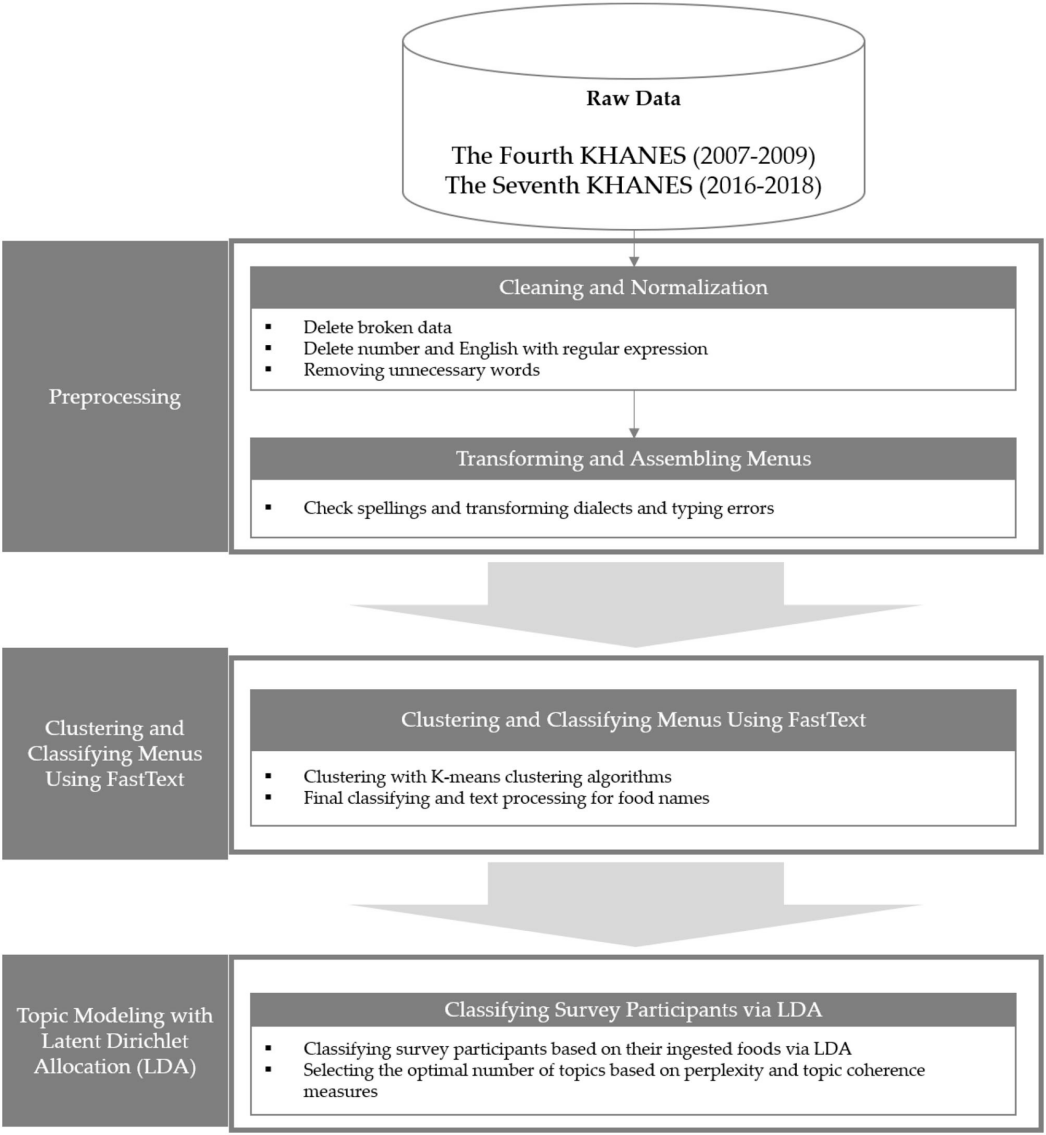
Data preprocessing process.

We preprocessed the two combined datasets: the 2007–2009 and 2016–2018 datasets. The preprocessing of these datasets is presented in Figure 1.

First, data with incorrect column values (e.g., food name in the weight column), which were distorted since they invaded columns on both sides, were deleted. Thus, 6,954 and 17,774 food names were extracted from each dataset. The vocabulary that emerged only once in the subsequent cleaning and normalization process through frequency analysis, was removed, not only because it does not affect the conduct of topic modeling but also because it is seen as an outlier. In addition, the differences in the surveyor's marking were unified and eliminated as much as possible by excluding the English characters, numbers, and special symbols contained in the extracted food names. For example, “Chocolett (marked in Korean pronunciation)” and “Chocolate” were unified as “Chocolate.” Thus, all food name data were tested for “Korean spelling” because of differences in spelling (dialects, etc.) or because the typos of each surveyor were not collected systematically. The Python package “Hanspell” based on the NAVER Korean Spellchecker was used for the Korean spelling test. Subsequently, 1,59,552 rows (87.86% of the raw 2007–2009 dataset) and 1,79,846 rows (78.89% of the raw 2016–2018 dataset) of data remained, and 1,178 and 1,653 food name data were used as experimental data. When the two food name data were merged, a total of 1,877 food name data were generated.

For the final food data, fastText, which is a methodology for embedding words developed on Facebook, was used to process food names for analysis. Word embedding involves replacing a word with a word vector containing meanings based on distributed simplicity-based presentation. fastText assumes that the peripheral words of a word with a similar distribution also have similar meanings. A word is represented as a vector with a continuous value in a predetermined dimension on the variance table. Word vectors created in this way contain their meaning and can also be used to analyze the operations and similarities between word vectors. fastText is an advantageous way to cluster similar words. It is another method that complements the disadvantage of hard-to-solve out-of-vocabulary (OOV) problems or unknown word problems. fastText is word-embedded for each *n-*gram of every word in the dataset and has the advantage of being able to calculate similarities to other words for OOV words that are unknown *via* known subwords.

**Figure 2:**
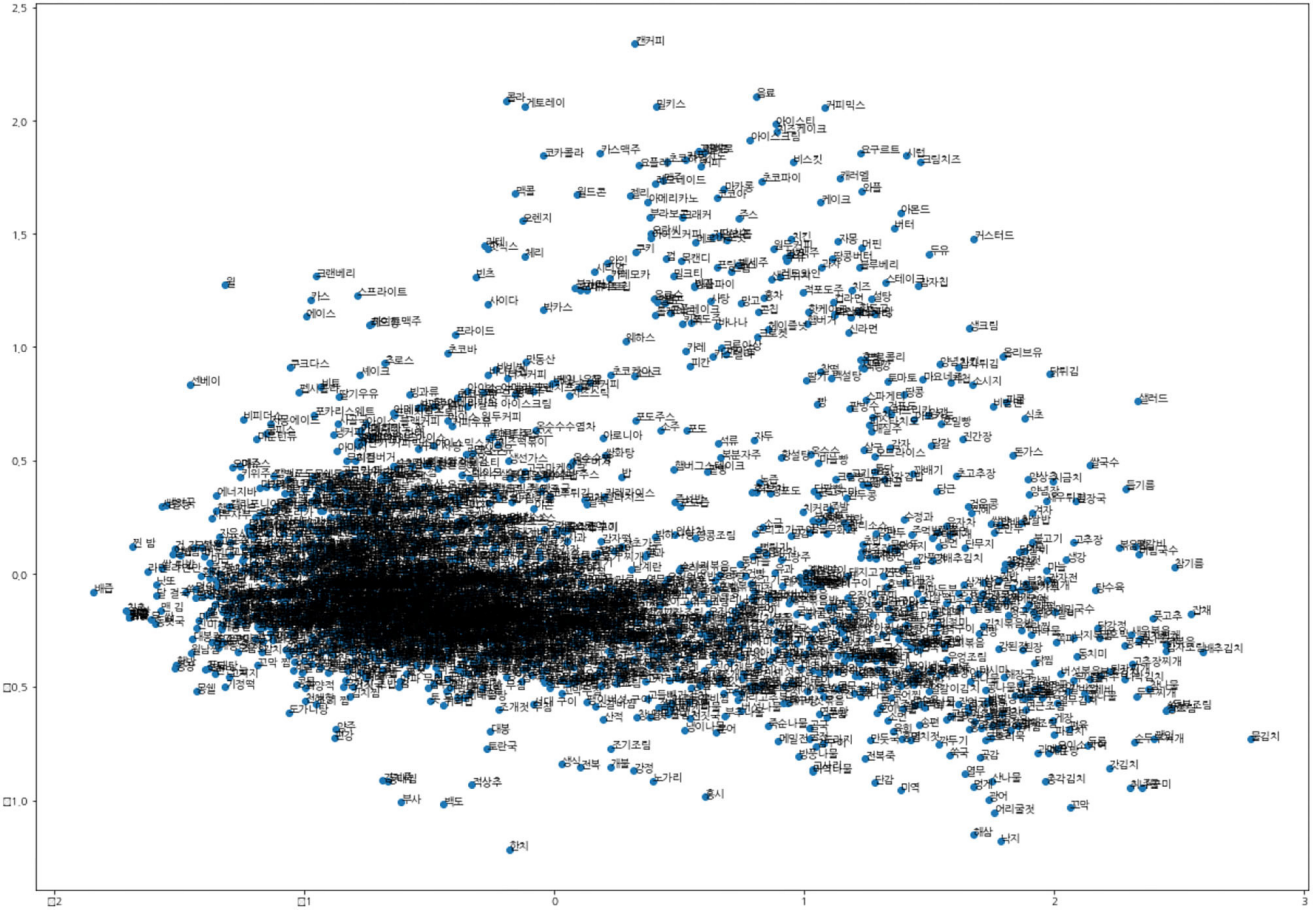
Two-dimensional scatter plot of food name vectors.

In this experiment, upon grouping the food names using fastText, the grouped foods' formations are similar. Thus, manual processing becomes cheap after our preprocessing procedures. This process is time-efficient and produces more accurate food processing data than by hand. To apply fastText, the Korean Wikipedia, KorQuad, and NAVER movie corpus were used to embed words using fastText as a morpheme-treated corpus. Since the Korean language can be broken down into alphabetical units, consonant-level learning using *MeCab_jamo* was conducted. The proportions of each word-embedding methodology were considered for both syllable and consonant units by treating them at a ratio of 50–50%, respectively. Figure 2 presents the two-dimensional scatter plot of food name vectors in this study using principal component analysis.

Final modifications were made to some heterogeneous food names in clusters using the designated cluster number of food names. As a result, 887 food names were derived from the 2007–2009 and 2016–2018 datasets. The final processed food names were treated with a weighted average of food divided by the food intake weight by certain survey participants, and the rounded values were treated with keyword-inclusion frequency.

#### Latent Dirichlet Allocation (LDA)

**Figure 3:**
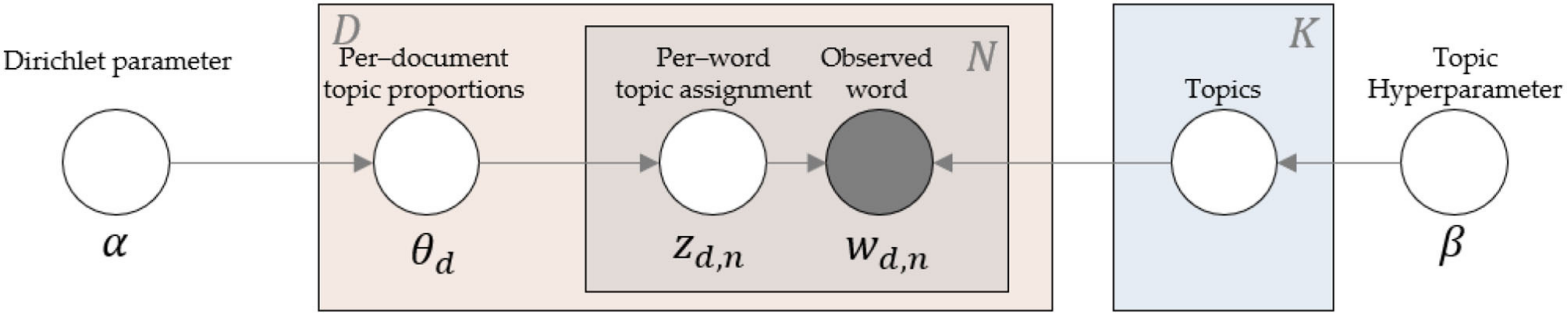
Intuition behind LDA.

Topic modeling methods are powerful, intelligent techniques widely applied in NLP to discover topics and semantic mining from unordered documents. LDA, one of the most popular topic modeling methods, is a generative probabilistic model for collecting discrete data, such as text corpora. It generates a topic per-document model and words per topic model using the Dirichlet distribution. Figure 3 presents the concept of LDA.

Many studies have applied LDA to topic modeling methods in various fields, such as keyword selection, source code analysis, opinion mining, event detection, music key profiling, image classification, a recommender system, sentiment classification, and even political issues (13).

In this study, we applied LDA based on the Gibbs sampling method owing to its rapid speed compared with the original model. Gibbs sampling is a Markov chain Monte Carlo algorithm for sampling conditional distributions of variables, approximated from an actual distribution when direct sampling is inefficient or difficult. Equation (1) expresses the updated LDA equation using Gibbs sampling for the probability that the *k-*th topic is assigned to *z*_*d, i*_, the *i-*th word of the *d-*th document.

(1) $$\begin{array}{r} p\left(z_{d,\;i}=k|z_{-i},\;\text{w}\right)=\frac{n_{d,\;k}+\alpha_{k}}{\sum_{i=1}^{K}n_{d,\;i}+\alpha_{i}}\frac{v_{k,\;w_{d,\;n}}+\beta_{w_{d,\;n}}}{\sum_{j=1}^{V}v_{k,\;j}+b_{j}}=\text{AB} \end{array}$$

In Equation (1), *z*_−*i*_ denotes leaving the *i-*th term from the calculation; **w**, the word vector of documents; *n*_*d, k*_, the number of times words in the *d-*th document were assigned to the *k-*th topic; *w*_*d, n*_, the *n-*th word in the *d-*th document; and *v*_*k*, *w*_*d, n*__, frequency of the word *w*_*d, n*_ from the whole corpus in the *k-*th topic. α_*k*_ and β_*k*_ are the hyperparameters of per-corpus topic distributions and per-document topic proportions, following symmetric Dirichlet distributions. Equation (1) can be summarized into two parts: **A** and **B**. **A** denotes the relationship between the *d-*th document and the *k-*th topic, and **B** indicates the relationship between the *n-*th words of the *d-*th document and the *k-*th topic.

To select the optimal number of topics for the LDA model, we considered perplexity and topic coherence measures: *C*_*V*_. Perplexity, generally used in language modeling, is the entropy-based measurement of the accuracy of sample prediction by a probability distribution or model. It is algebraically equal to the inverse of the geometric mean per word likelihood. The *C*_*V*_ measure is based on a sliding window, one-set segmentation of the top words, and an indirect confirmation measure that uses normalized pointwise mutual information and cosine similarity.

Moreover, before conducting LDA, we preprocessed the survey data, which were unstructured and unrefined text data, to develop a more elegant and time-saving method than modification by hand. We cleaned and corrected over 4,00,000 food name data in 5 min. Also, we vectorized the food name and clustered them using the K-means clustering algorithm. Finally, we refined the coherent food name data rapidly. These novel methods for preprocessing raw data used in this study are significant to future researchers who will be using food consumption data, such as KNHANES data. Since they should preprocess before analyzing them, our preprocessing method can be employed as an essential process for systematically applying the researchers' methodologies ranging from traditional methods to machine learning studies with high accuracy.

## Results

**Figure 4:**
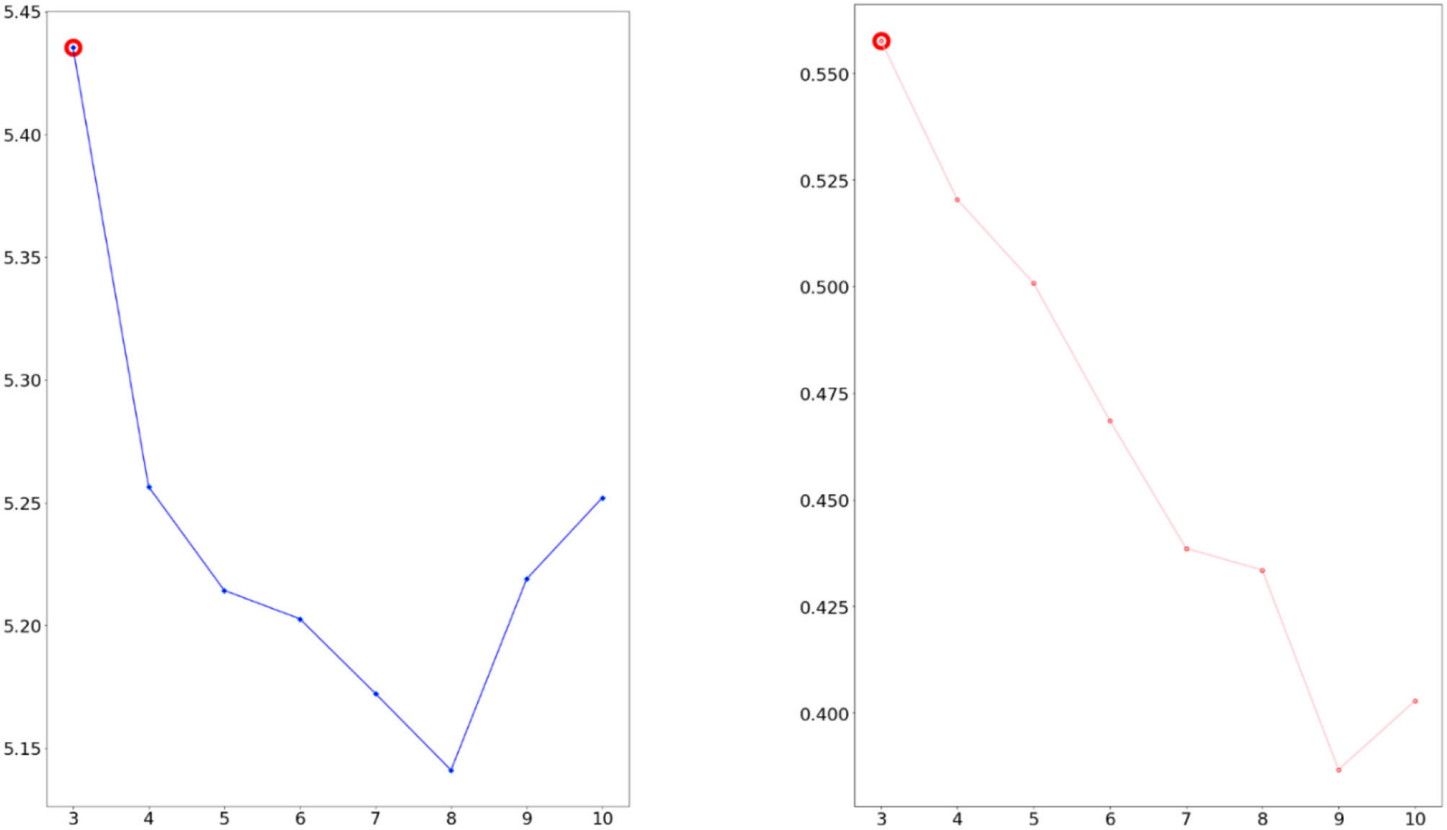
Calculated perplexity (left) and **C**_**V**_ (right) for selecting optimal number (2007–2009 dataset).

**Figure 5:**
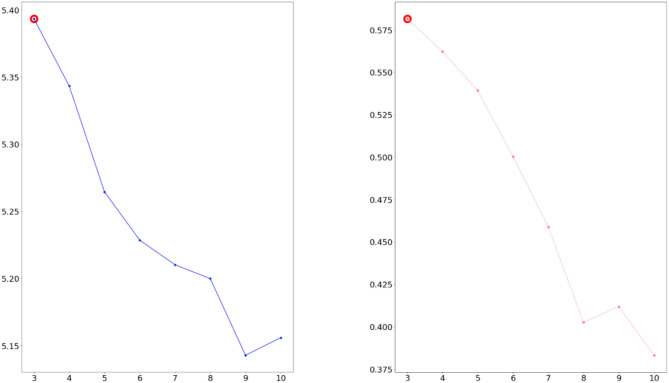
Calculated perplexity (left) and **C**_**V**_ (right) for selecting optimal number (2016–2018 dataset).

We considered the five indicators of perplexity and coherence score *C*_*V*_. If two measures have higher values, the data were classified better. As a result, we confirmed three topics as the optimal topic number from Figures 4, 5.

Furthermore, we extracted three dietary patterns from 2007 to 2009 and 2016 to 2018, respectively. We illustrated the three dietary patterns extracted from the 2007–2009 dataset and showed that the top 10 frequently appearing food names belong to those patterns, as shown in Table 2. Topic 1 consists of “Americano,” “kimchi,” “white rice,” “egg,” “multigrain rice,” and so on. Topic 2 contains “instant coffee,” “white rice”, “ramen,” “kimchi,” “pumpkin,” and so on. Lastly, Topic 3 comprises “soybean paste soup (doenjang soup),” “mix of red pepper paste and soybean paste (ssamjang),” “cabbage,” “kimchi,” “white rice,” and so on.

**Table 2:** Topic-based dietary patterns of 2007–2009.

**Rank**	**Topic 1**	**Topic 2**	**Topic 3**
	**(Token: 51.3%)**	**(Token: 25.0%)**	**(Token: 23.7%)**
	**(Participant: 68.4%)**	**(Participant: 16.2%)**	**(Participant: 15.3%)**
1	Americano	Instant coffee	Soybean paste soup (Doenjang Soup)
2	Kimchi	White rice	Mix of red pepper paste and soybean paste (Ssamjang)
3	White rice	Ramen	Cabbage
4	Egg	Kimchi	Kimchi
5	Multigrain rice	Pumpkin	White rice
6	Stir-fried anchovy	Cutlassfish	Lettuce
7	Bean sprout	Cucumber	Garlic
8	Sweet potato	Snack	Sesame leaf
9	Marinated meat (Jangjorim)	Cabbage	Red pepper
10	Spinach	Beer	Multigrain rice

These topics are similar to traditional Korean dietary patterns, consisting mainly of rice, soup, kimchi, vegetables, and fish. Each topic accounts for 68.4, 16.2, and 15.3% of the survey participants, respectively. Also, the token proportions of dietary patterns (the number of food names that appeared in each topic) were 51.3, 25.0, and 23.7%, respectively. Similarly, we extracted t three dietary patterns from the 2016 to 2018 dataset and showed that the top 10 frequently appearing food names belong to those shown in Table 3. Topic 1 consists of “red chili and soybean paste (ssamjang),” “pork belly,” “lettuce,” “red pepper,” “cold noodle (naengmyeon),” and so on. Topic 2 contains “kimchi,” “instant coffee,” “milk,” “white rice,” “multigrain rice,” and so on. Lastly, Topic 3 comprises “americano,” “fried chicken,” “mayonnaise,” “fish cake soup,” and so on.

**Table 3:** Topic-based dietary patterns of 2016–2018.

**Rank**	**Topic 1**	**Topic 2**	**Topic 3**
	**(Token: 15.0%)**	**(Token: 62.3%)**	**(Token: 22.7%)**
	**(Participant: 17.4%)**	**(Participant: 67.1%)**	**(Participant: 15.5%)**
1	Mix of red pepper paste and soybean paste (Ssamjang)	Kimchi	Americano
2	Pork belly	Instant coffee	Fried chicken
3	Lettuce	Milk	Mayonnaise
4	Red pepper	White rice	Fish cake soup
5	Cold noodle (Naengmyeon)	Multigrain rice	Ramen
6	Onion	Soybean paste soup (Doenjang Soup)	Snack
7	Soju	Kimchi stew	Chicken breast
8	Grilled mushrooms	Apple	Soda
9	Orange juice	Roasted seaweed	Sausage
10	Duck meat	Stir-fried anchovy	Beer

Each topic accounts for 17.4, 67.1, and 15.5% of the survey participants, respectively. Moreover, the token proportions of dietary patterns (the number of food names that appeared in each topic) were 15.0, 62.3, and 22.7%, respectively.

The main changes in the dietary patterns of the 2016–2018 dataset compared with that of the 2007–2009 dataset are as follows:

First, an unhealthy dietary pattern occurred in Topic 1 of the 2016–2018 dataset, leading to “americano—fried chicken—ramen—snack—soda—beer,” which accounts for 15.5% of the survey participants. Second, the new dietary pattern represented as meat and alcohol-oriented dining out pattern occurred in Topic 2, “mix of red pepper paste and soybean paste (ssamjang)—pork belly—lettuce—pepper—cold noodle—onion—soju,” accounting for 17.4% of the survey participants.

**Figure 6:**
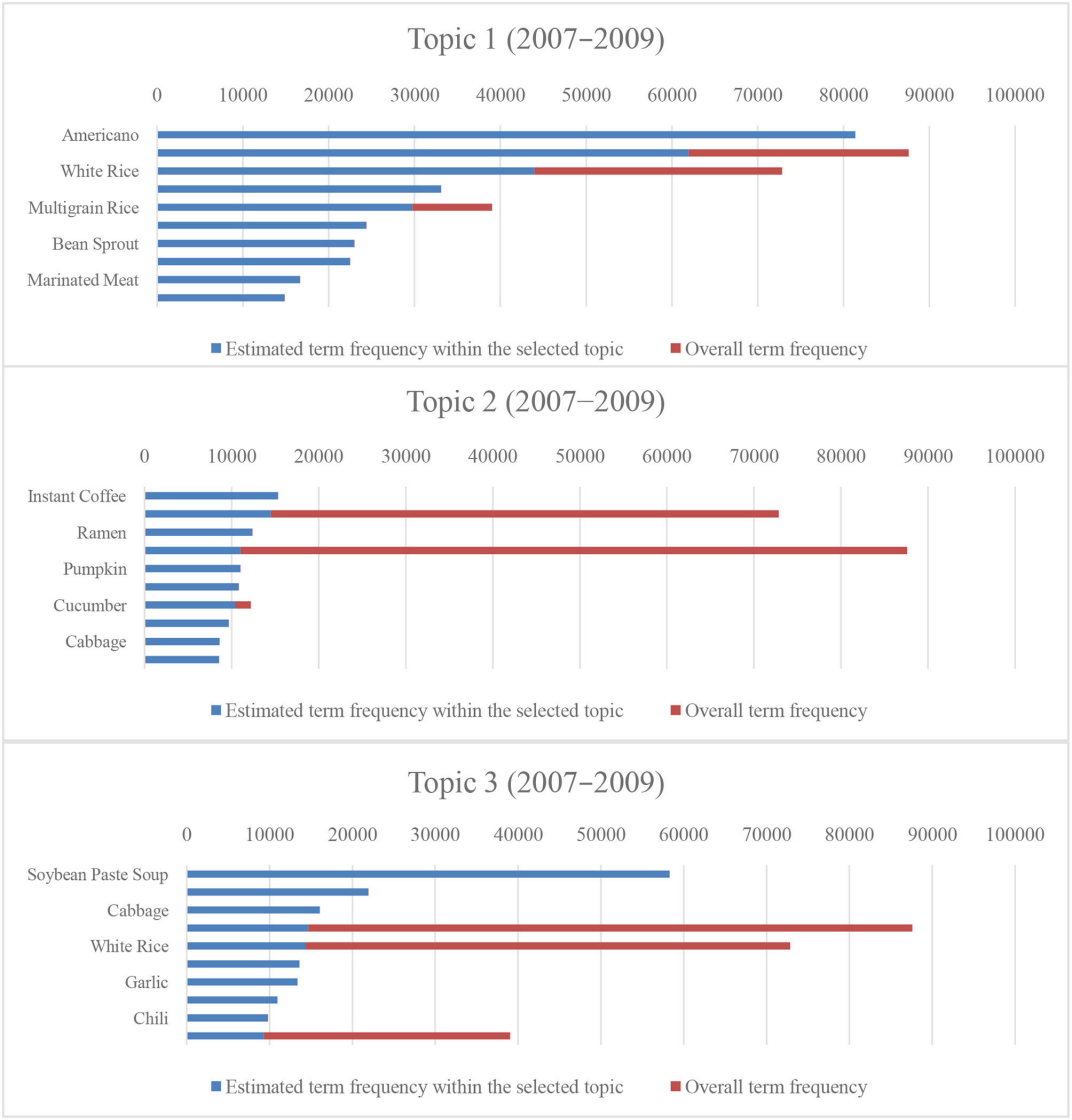
Number of tokens and proportions of entire tokens for the top 10 menus (2007–2009).

**Figure 7:**
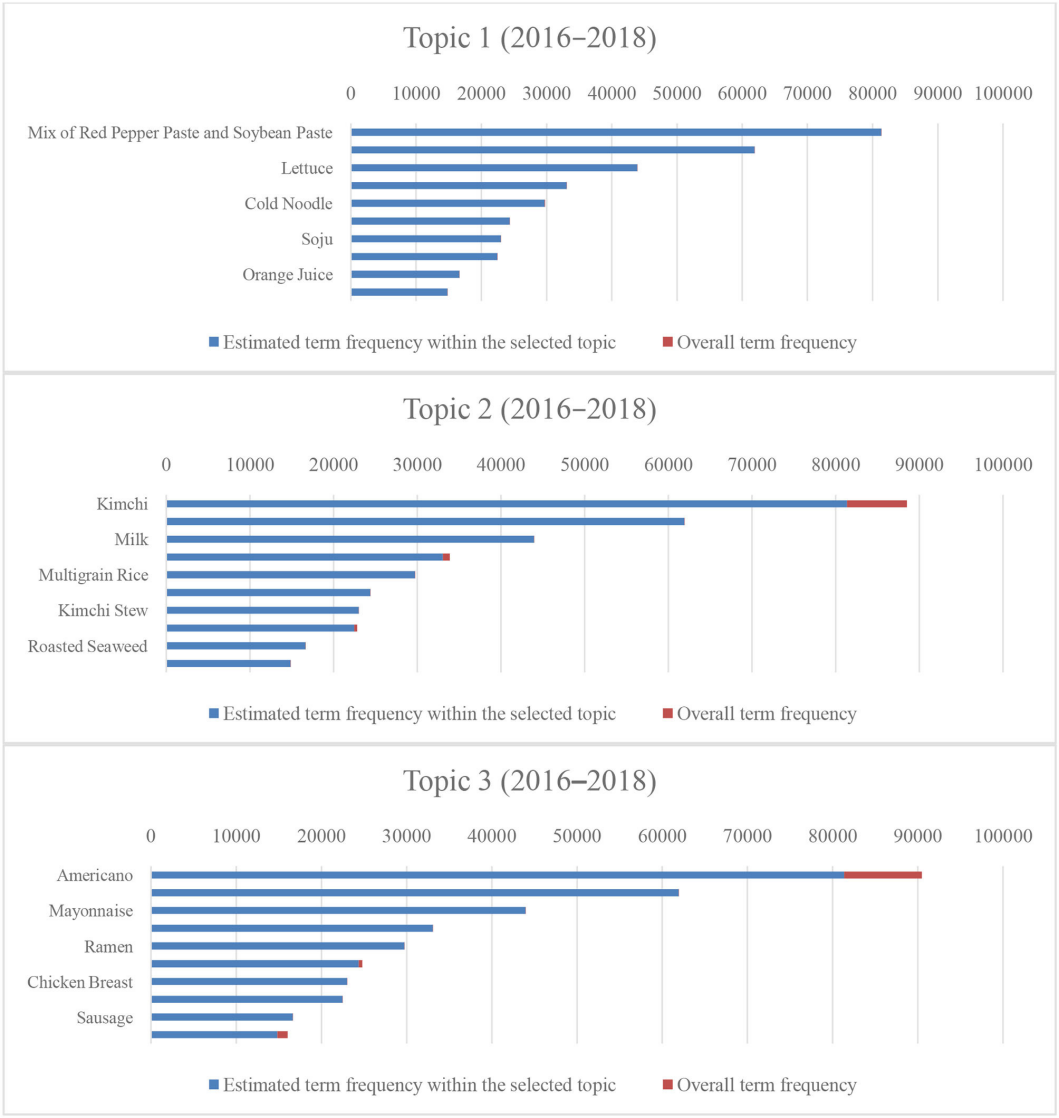
Number of tokens and proportions of entire tokens for the top 10 menus (2016–2018).

Figures 6, 7 present the estimated term frequency within the selected topics and the overall term frequency of our results. Suppose the difference between the overall and estimated term frequencies within the selected topic becomes smaller, the uniqueness of the data in the topic becomes larger. In other words, the proportion of biased people toward such a diet increases. Following the previous sentences, as the topic classification for 2016–2018 differs, the diet of the survey participants was more clearly divided between 2016 and 2018. The result indicates that the difference in the dietary patterns rapidly yielded unhealthy dietary patterns, including meat and the aforementioned alcohol-oriented dietary pattern.

**Figure 8:**
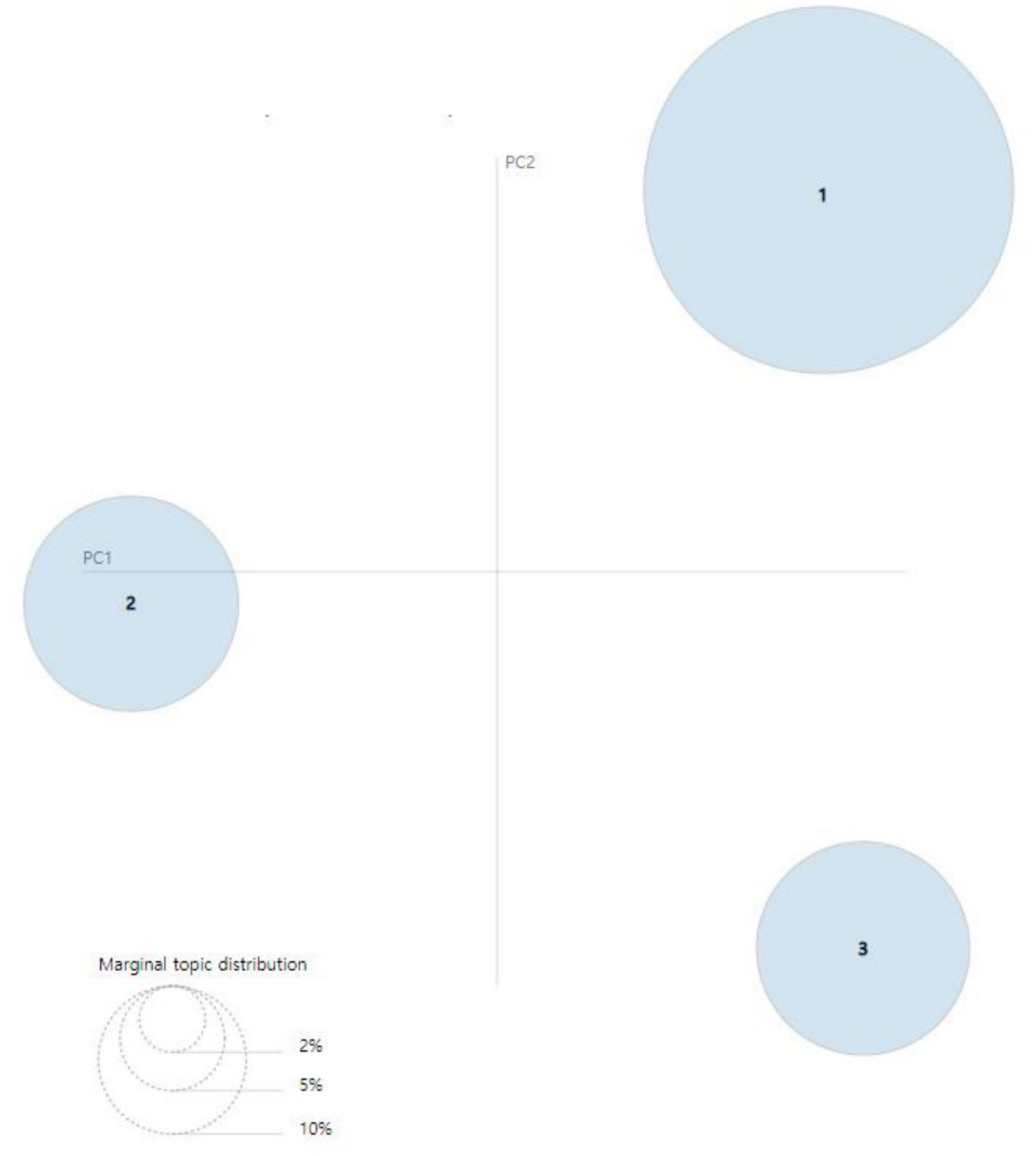
Visualization results of inter-topic distance map *via* multidimensional scaling (MDS) (2007–2009) (14).

**Figure 9:**
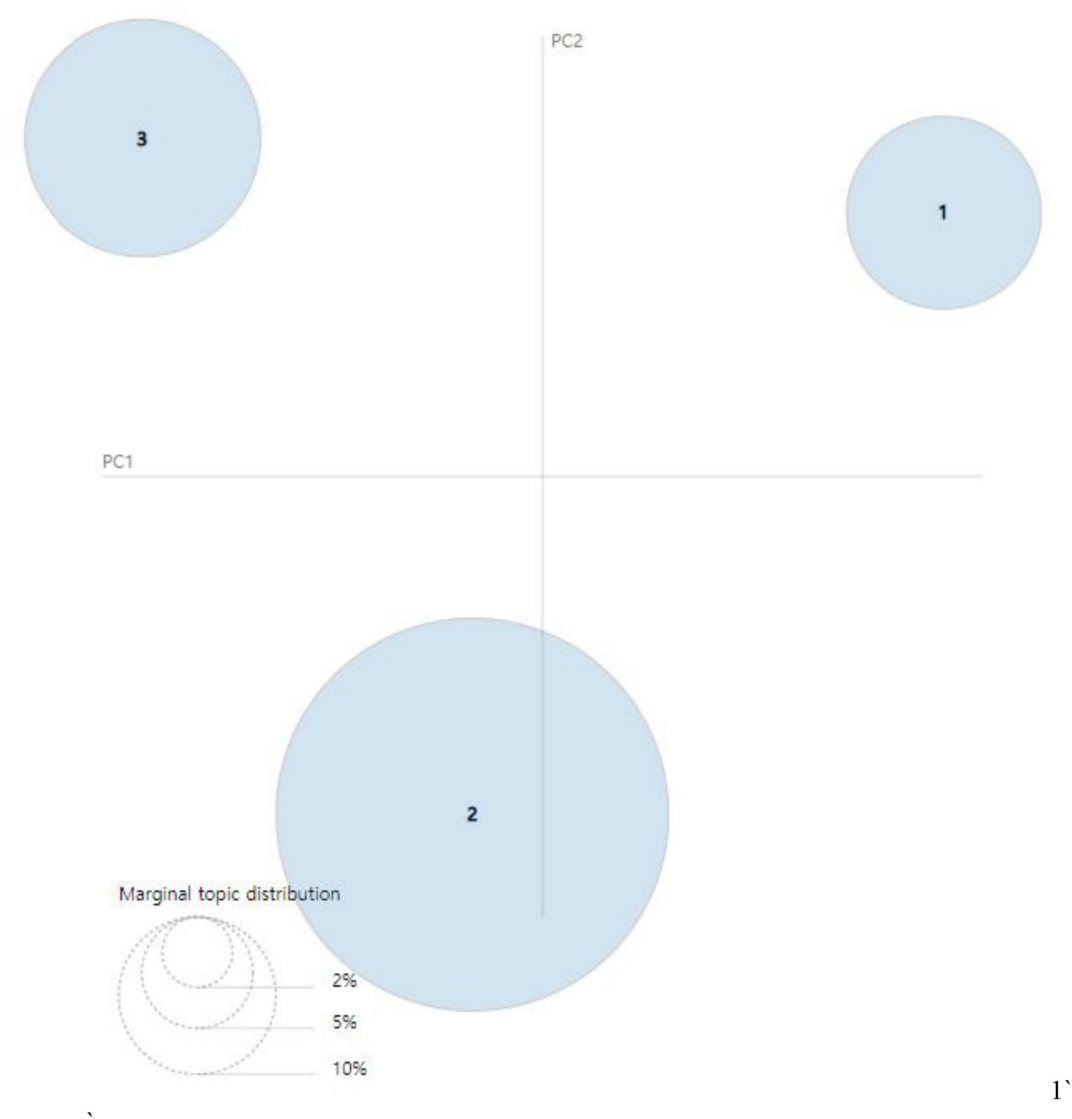
Visualization results of inter-topic distance map *via* multidimensional scaling (MDS) (2016–2018) (14).

Figures 8, 9 present the intertopic distances onto two dimensions from the final topic classification results. The figures originated from topics as circles in the two-dimensional plane, whose centers are determined by computing the Jensen–Shannon divergence (JSD) between topics and then using multidimensional scaling to project the intertopic distances onto two dimensions. Each topic's overall prevalence is encoded using the areas of the circles. The JSD is a method for measuring the similarity between two probability distributions. It is also known as the information radius (IRad) or total divergence to the average. Its square root is a metric referred to as the Jensen–Shannon distance (15, 16). The formula of the JSD is as follows:

Consider the set $M_{+}^{1}\left(A\right)$ of probability distributions, where *A* denotes a set provided with some σ-algebra of measurable subsets. In particular, let A be a finite or countable set with all subsets being measurable. Then, the JSD $M_{+}^{1}\left(A\right)\times M_{+}^{1}\left(A\right)\to \left[0,\;\infty \right)$ is a symmetrized and smoothed version of the Kullbeck–Leibler divergence *D*(*P* ∥ *Q*). It is defined by $JSD\left(P∥Q\right)=\frac{1}{2}\text{D}(\text{P}∥M)+\frac{1}{2}$D(Q ∥ *M*), where $M=\frac{1}{2}\left(P+Q\right)$. The geometric JSD yields a closed form for divergence between two normal distributions by taking their geometric mean. The more general definition that allows comparison of more than two probability distributions is as follows:

(2) $$\begin{array}{r} JSD_{\pi_{1},\;{\;\pi }_{2},\;\ldots ,\;{\;\pi }_{n}}\left(P_{1},\;P_{2},\;\ldots ,\;P_{n}\right)=\overset{n}{\underset{i=1}{\sum }}\pi_{i}D\left(P_{i}∥M\right)\\ \;=H\left(\overset{n}{\underset{i=1}{\sum }}\pi_{i}P_{i}\right)-\overset{n}{\underset{i=1}{\sum }}\pi_{i}H(P_{i}) \end{array}$$

where $M\overset{n}{\underset{i=1}{\sum }}\pi_{i}P_{i}$; π_1_, π_2_, …, π_*n*_ are selected weights from the probability distributions *P*_1_, *P*_2_, …, *P*_*n*_; and *H*(*P*) is the Shannon entropy for distribution P. To calculate JSD for illustrating the inter-topic map, we take *P*_1_ = *P, P*_2_ = *Q*, $\pi_{1}=\pi_{2}=\frac{1}{2}$. Thus, for distributions *P* and *Q*, the JSD is as follows:

(3) $$\begin{array}{r} JSD=H\left(M\right)-\frac{1}{2}\left(H\left(P\right)+H\left(Q\right)\right) \end{array}$$

## Discussion

In a large-scale national survey data, we found three unique Korean dietary patterns from the 2007–2009 and 2016–2018 datasets using AI methodologies. By applying LDA, we observed changes in the Korean dietary patterns over the past decade. In the 2007–2009 dataset, the three major dietary patterns were composed of rice, soup, various vegetables, and fish, which is a traditional dietary pattern. However, in the 2016–2018 dataset, Western dietary patterns based on meat and alcohol (Topic 1), or consisting of fried, processed foods and sugar-sweetened beverages (Topic 3), and a traditional dietary pattern (Topic 2) were observed.

Dietary patterns and changes in food intake among Korean adults were similar to those from previous studies (4, 11, 12, 17). Studies using the KNHANES dataset examined during the same period demonstrated that the major dietary patterns composed of white rice and Kimchi, or grains, vegetables, and fish were extracted using factor analysis (11, 17). Studies using the fifth KNHANES dataset reported the meat and alcohol patterns using a traditional method for dietary pattern extraction (12). Kweon et al. (18) reported a decrease in the intake of grains, vegetables, and carbohydrates and an increase in the intake of beverages (sugar-sweetened beverages), meat, dairy, eggs, and fat. Furthermore, they reported an increase in the intake of snacks, frequency of dining out, and consumption of convenience foods over the past 20 years from the KNHANES datasets between 1998 and 2018, which have similar trends to our results.

Over the last decade, the dietary patterns have changed from traditional to unhealthy patterns among Korean adults. These changes may be influenced by Western diets, and an increase in the frequency of eating out, particularly in young Korean adults. Western dietary patterns are characterized by high intakes of meat, fried foods, refined grains, and added sugars, which may produce unfavorable health outcomes (19–22). Frequent eating out is associated with a higher intake of total energy, fat, saturated fat, cholesterol, and sodium, and a lower intake of dietary fiber (4, 23). Therefore, the shift in dietary patterns should be continuously monitored, and whether the associated changes produce healthy outcomes among Koreans should be checked.

Our findings confirm that the AI and NLP techniques produce similar results to the traditional method previously used for dietary pattern extraction, suggesting that the current NLP-based method can be widely used in nutrition epidemiology.

Our proposed LDA method offers some advantages compared with the traditional method. First, the LDA method provides more specific information on the composition of dietary patterns. For example, in the meat and alcohol pattern (Topic 1), a mix of red pepper and soybean pastes (ssamjang), pork belly, lettuce, red pepper, cold noodle (naengmyeon), onions, and soju is a typical Korean menu for dining out. This result indicates that the recent increase in this dietary pattern compared with 10 years ago may reflect an increase in the frequency of dining out in Korean adults.

However, a traditional method does not provide details about the composition of dietary patterns. Previous studies extracted “meat and alcohol patterns” but did not capture the meat or alcohol types (17). The reason why a traditional method cannot depict details is that the method depends on food item classifications, which vary depending on the investigators (12, 24, 25). For text preprocessing, we developed the semiautomatic methodology for preprocessing unstructured data, such as dietary recorded text data. Furthermore, we developed the preprocessing method using fastText, the unsupervised or supervised learning algorithm, for obtaining vector representations for words. This method rapidly reduces the preprocessing time for preparing experiments for extracting dietary patterns. The LDA method for classifying topics based on frequently appearing words obtains useful nutritional or dietary information that cannot be obtained by traditional dietary pattern extraction methods. We found that Korean adults consumed pork belly and soju frequently using the LDA method. An advantage of the LDA technique is that it needs no assumption to derive dietary patterns (26–28). Without a clue on the dietary pattern, LDA can extract the dietary patterns using the probabilistic model. Furthermore, it is easy to use, less labor-intensive, and more efficient than the traditional method. For instance, suppose more data are added or a new survey is generated, we can easily extend the data, find new patterns *via* topics, and compare them based on frequently appearing data (28–31).

## Conclusion

In this study, we proposed a novel NLP for extracting dietary patterns. Our results indicate that dietary patterns among Korean adults have evolved from traditional to more Western dietary patterns over the past decade. Given the efficiency and validity of the NLP methods, we recommend that the current NLP-based method be applied in nutrition epidemiology.

Our methodologies and results can contribute to enabling diet research using unstructured data more quickly. In addition, the diary pattern can be identified from a new perspective by performing a nonlinear diary pattern classification based on machine learning rather than a linear dietary pattern classification. Also, the strength of this study is in the application of the novel method using NLP to extract dietary patterns and obtain more useful dietary information, unlike traditional dietary pattern extraction methods. Our results suggest the potential of applying AI-based methods in various fields of nutritional mechanics.

However, the limitations of our research are as follows: First, the experiment results may differ because of the text preprocessing procedure. Second, the last food groups for extracting dietary patterns include subjective opinions of researchers. This means if we set the food groups in different ways, then the conclusion may differ. Therefore, if we improved those limitations, then a more computationally clear and objective dietary pattern may be derived.

Further research on the association between dietary patterns and disease risk can be attempted using machine-learning-based methodologies. In addition, pretrained models for the NLP food name datasets in the nutrition area should be developed.

## Data Availability Statement

Publicly available datasets were analyzed in this study. This data can be found here: https://knhanes.kdca.go.kr/knhanes/eng/index.do.

## Ethics Statement

Ethical review and approval was not required for the study on human participants in accordance with the local legislation and institutional requirements. The patients/participants provided their written informed consent to participate in this study.

## Author Contributions

WK and JK: conceptualization and project administration. IC: methodology, software, formal analysis, and visualization. IC and WK: data curation and supervision. IC and JK: writing—original draft preparation and writing—review and editing. All authors have read and agreed to the published version of the manuscript.

## Funding

This work was supported by the National Research Foundation of Korea (NRF) grant funded by the Korean Government (Ministry of Science and ICT) (No. 2021R1A2C1003211, to JK).

## Conflict of Interest

The authors declare that the research was conducted in the absence of any commercial or financial relationships that could be construed as a potential conflict of interest.

## Publisher's Note

All claims expressed in this article are solely those of the authors and do not necessarily represent those of their affiliated organizations, or those of the publisher, the editors and the reviewers. Any product that may be evaluated in this article, or claim that may be made by its manufacturer, is not guaranteed or endorsed by the publisher.
